# Shikonin promotes autophagy in BXPC-3 human pancreatic cancer cells through the PI3K/Akt signaling pathway

**DOI:** 10.3892/ol.2014.2293

**Published:** 2014-06-26

**Authors:** SHUQING SHI, HAIMEI CAO

**Affiliations:** Department of General Surgery, Third Xiangya Hospital, Central South University, Changsha, Hunan 410013, P.R. China

**Keywords:** shikonin, BXPC-3 cells, autophagy, LC3

## Abstract

The present study aimed to investigate the effect of shikonin on autophagy in BXPC-3 human pancreatic cancer cells and its underlying mechanism. Cell viability was assessed using the Cell Counting Kit-8 assay and the expression of light chain (LC) 3, p62, phosphoinositide 3-kinase (PI3K), Akt, phosphorylated (p)-PI3K and p-Akt was analyzed using western blot analysis. Following treatment with 1 μmol/l shikonin for 48 h and 2.5 and 5 μmol/l shikonin for 24 and 48 h, the viability of the BXPC-3 cells was found to be significantly reduced and the protein expression of LC3-II/LC3-I was observed to be increased, while the protein expression of p62, PI3K, Akt, p-PI3K and p-Akt was decreased. These findings suggest that shikonin promotes autophagy in BXPC-3 cells and that the underlying mechanism may be associated with the PI3K/Akt signaling pathway.

## Introduction

Pancreatic cancer is one of the most common malignant tumors of the digestive system due to its latent onset, difficulty in surgical resection, poor prognosis and high mortality rate, for which the only effective treatment at present is surgical resection. Due to the lack of efficient methods for the early diagnosis of pancreatic cancer, as well as its highly malignant nature and potential for metastatic progression during its early stages, only 10% of all pancreatic cancer patients present with resectable disease at diagnosis (1). Autophagy is a process that facilitates self-digestion and degradation of proteins and organelles in eukaryotic cells, which affects the incidence of tumors, neurodegeneration and myocardial hypertrophy (2). Previous studies have reported that autophagy also leads to cell death, a nonapoptotic form of programmed cell death, which is termed autophagic or type II cell death to distinguish it from apoptosis (3,4). LC3 and p62 are molecular markers of autophagy, in which the ratio of LC3-II to LC3-I increases, while the expression of p62 decreases (5). The PI3K/Akt signaling pathway has an important role in the regulation of autophagy (6); however, the specific signaling mechanism underlying autophagy has yet to be elucidated. Thus, PI3K/Akt signaling molecules may have potential therapeutic targets in pancreatic cancer (7).

In recent years, a number of studies have aimed to extract and screen the active components from traditional Chinese herbs in order to develop more effective drugs for cancer treatment (8,9). Shikonin, a naphthoquinone derived from the roots of lithospermum, has been reported to inhibit the proliferation of tumor cells in breast, liver and lung cancer, as well as induce cell death through apoptosis and cytoclasis (10–12). However, the effect of shikonin on autophagy has yet to be elucidated. The present study aimed to investigate the effect of shikonin on autophagy in BXPC-3 human pancreatic cancer cells, as well as its underlying mechanism.

## Materials and methods

### Reagents

**Figure 1:**
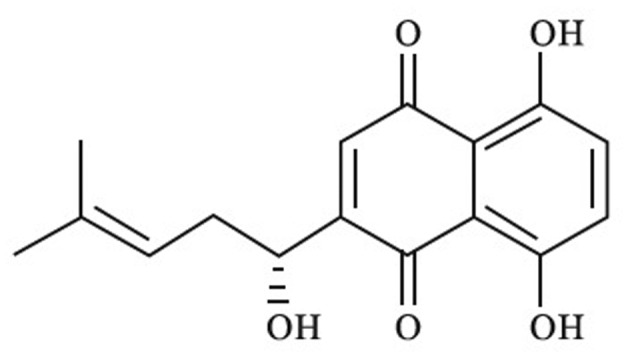
Chemical structure of shikonin.

Shikonin was purchased from the Guangzhou Institute for Drug Control (Guangzhou, China). The chemical structure of shikonin is shown in Fig. 1. All other chemicals and reagents were purchased from Sigma-Aldrich (St. Louis, MO, USA), unless specified otherwise.

### Cell culture

The BXPC-3 pancreatic cancer cell line was obtained from the Institute of Biochemistry and Cell Biology, Chinese Academy of Sciences (Shanghai, China) and cultured in RPMI-1640 medium supplemented with 10% fetal calf serum (HyClone Laboratories, Inc., Logan, UT, USA), penicillin and streptomycin at 37°C in 5% CO_2_. Cells were regularly passaged to maintain exponential growth.

### Cell proliferation assay

Proliferation of the BXPC-3 cells was analyzed using the Cell Counting Kit-8 (CCK8; Tongren Shanghai Co, Shanghai, China) according to the manufacturer’s instructions. In brief, the BXPC-3 cells were washed, counted and seeded at a density of 4×10^5^ cells/ml in each well on 96-well plates. After 6 h, specific concentrations of shikonin (0, 1, 2.5 and 5 μmol/l) were added to the cells. At 24 and 48 h after treatment, CCK8 solution was added and incubated for 4 h. Cell viability was determined using a spectrophotometer (NanoDrop 1000, Thermo Fisher Scientific, Waltham, MA, USA) at an absorbance of 450 nm. The viability of the cells was calculated according to the following formula: Cell viability (%) = 100 − ((mean absorbance of untreated group − mean absorbance of treatment group)/mean absorbance of untreated group ×100). Results were calculated based on three individual experiments performed in triplicate.

### Western blot analysis

Cells were collected and lysed using radioimmunoprecipitation assay lysis buffer (Santa Cruz Biotechnology, Inc., Santa Cruz, CA, USA). The cell lysates were collected following centrifugation and the protein concentration was quantified using a bovine serum albumin (BSA) method. Equal quantities of protein were loaded and separated using 10% SDS-PAGE, then transferred to nitrocellulose membranes (Millipore Corporation, Billerica, MA, USA). The membranes were blocked with 3% BSA for 2 h, then incubated with polyclonal rabbit anti-mouse LC3, polyclonal rabbit anti-mouse p62, polyclonal rabbit anti-mouse PI3K, polyclonal rabbit anti-human p-PI3K, polyclonal rabbit anti-rat Akt, polyclonal rabbit anti-rat p-Akt and polyclonal rabbit anti-rat β-actin antibodies (Santa Cruz Biotechnology, Inc.) at 4°C overnight. Subsequent to washing three times with Tris-buffered saline and Tween 20, the membranes were incubated with corresponding horseradish peroxidase-conjugated polyclonal goat anti-rabbit secondary antibodies (Santa Cruz Biotechnology, Inc.) for 1 h at room temperature. The membranes were developed using an enhanced chemiluminescence kit (Santa Cruz Biotechnology, Inc.) and exposed to X-ray film. β-actin was used as a loading control. The density of the bands on the membrane was analyzed using an image analyzer (LabWorks Software, Upland, CA, USA).

### Statistical analysis

Results are presented as the mean ± standard deviation. Statistical analyses were performed using Student’s t-test or one- or two-way analysis of variance followed by Tukey’s test. P<0.05 was considered to indicate a statistically significant difference.

## Results

### Effect of shikonin on BXPC-3 cell viability

**Figure 2:**
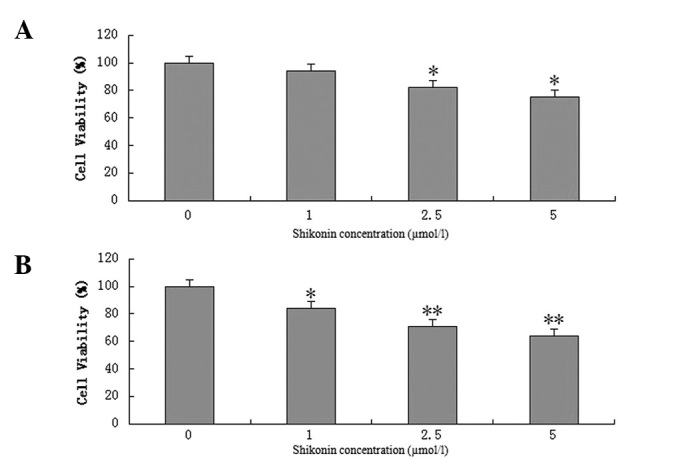
BXPC-3 cell viability detected using the Cell Counting Kit-8 assay following treatment with shikonin (0, 1, 2.5 and 5 μmol/l) after (A) 24 h and (B) 48 h. ^*^P<0.05 and ^**^P<0.01, vs. the control group.

BXPC-3 cells were exposed to various concentrations of shikonin (1, 2.5 and 5 μmol/l) for 24 and 48 h. Shikonin was found to have a dose-dependent effect on BXPC-3 cell viability (Fig. 2). After 24 h, 1 μmol/l shikonin was not observed to inhibit breast cancer cell growth; however, when the concentration was increased to 2.5 and 5 μmol/l, cancer cell survival was found to significantly decrease. After 48 h, 1, 2.5 and 5 μmol/l shikonin were observed to significantly inhibit BXPC-3 cell viability, and cancer cell survival decreased in a dose-dependent manner.

### Western blot analysis

**Figure 3:**
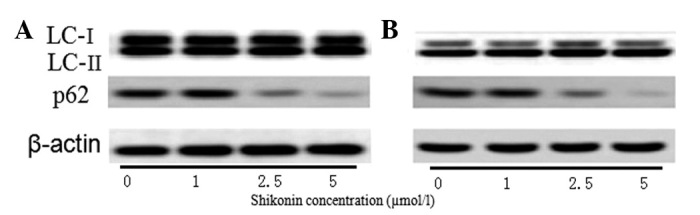
Effect of shikonin on the expression of LC3-I/LC3-II and p62 in BXPC-3 cells after (A) 24 h and (B) 48 h. LC, light chain.

The protein expression of LC3 and p62 was assessed using western blot analysis. In the cells treated with 2.5 and 5 μmol/l shikonin for 24 h, the protein expression of LC3-II/LC3-I was observed to increase, while that of p62 was found to decrease, compared with the serum-free positive control cells (Fig. 3A). After 48 h, the expression of LC3-II/LC3-I was observed to increase in the cells treated with 1, 2.5 and 5 μmol/l shikonin, while the expression of p62 was found to decrease (Fig. 3B).

**Figure 4:**
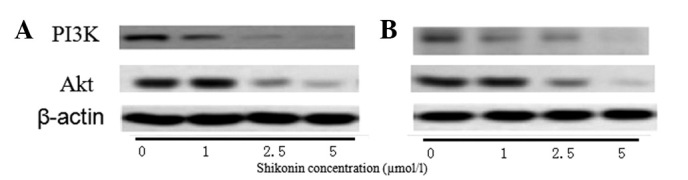
Effect of shikonin on the expression of PI3K and Akt in BXPC-3 cells after (A) 24 h and (B) 48 h. PI3K, phosphoinositide 3-kinase.

After 24 h (Fig. 4A), 1 μmol/l shikonin was not found to affect the expression of PI3K and Akt; however, at 48 h (Fig. 4B), the expression of PI3K and Akt were observed to decrease compared with that in the control cells. In the cells treated with 2.5 and 5 μmol/l, the expression of PI3K and Akt was found to decrease after 24 and 48 h in a dose-dependent manner.

**Figure 5:**
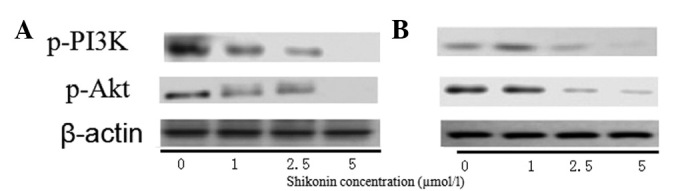
Effect of shikonin on p-PI3K and p-Akt expression in BXPC-3 cells after (A) 24 h and (B) 48 h. PI3K, phosphoinositide 3-kinase; p-, phosphorylated.

The protein expression of p-PI3K and p-Akt was also assessed using western blot analysis, which showed that shikonin affected the expression of p-PI3K and p-Akt in a dose-dependent manner. After 24 h (Fig. 5A), no significant difference was observed in the expression of p-PI3K and p-Akt in the cells treated with 1 μmol/l shikonin compared with that in the control cells. However, in the cells treated with 2.5 and 5 μmol/l shikonin, the expression of p-PI3K and p-Akt was found to be decreased compared with that in the control cells. After 48 h (Fig. 5B), compared with the control group, the expression of p-PI3K and p-Akt was observed to be decreased in all three treatment groups.

## Discussion

Pancreatic cancer is a highly prevalent malignant tumor of the digestive tract, which has a low survival rate. In general, the median survival time is three to five months and the five-year survival rate is <5% (13). Autophagy has been proposed to be an important survival pathway associated with the prevention and treatment of tumors (14,15). Autophagy is a highly conserved biological process in which a number of macromolecules participate in the degeneration and recycling of organelles. Autophagy is rare under normal conditions, but significantly increases during certain situations, including hunger, growth factor deficiency, oxygen deficiency, intracellular stress and growth signal stimulation. LC3 and SQSTM1/p62 are important proteins in autophagy, during which cytoplasmic-pattern LC3 (LC3-I) is converted to autophagosomal membrane LC3 (LC3-II), leading to increased LC3-II levels. However, p62 expression is generally decreased during autophagy (16). The PI3K/Akt/mammalian target of rapamycin (mTOR) signaling pathway is a key regulator of physiological cellular processes that are associated with proliferation, differentiation, apoptosis, motility, metabolism and autophagy (17). During hunger and oxygen deficiency, the PI3K/Akt signaling pathway has been reported to negatively regulate autophagy through mediating the expression of mTOR (18).

Shikonin, a naphthoquinone derived from the roots of lithospermum or cultured through plant tissue cultivation methods, inhibits breast, liver, rectum, mouth, bladder and skin cancer cells (19). The present study aimed to investigate the effect of varying concentrations of shikonin on the viability and autophagy of BXPC-3 pancreatic cancer cells. Shikonin was found to reduce BXPC-3 cell viability through increasing the expression of LC3-II/LC3-I and decreasing the expression of SQSTM1/p62 to promote BXPC-3 cell autophagy. In addition, the expression of PI3K and Akt, as well as the levels of p-PI3K and p-Akt were observed to decrease. These findings suggest that shikonin promotes autophagy through inhibiting the expression and phosphorylation of PI3K and Akt, as well as through inhibiting viability in BXPC-3 cells. A previous study on the antitumor effects of shikonin reported its role in accelerating apoptosis and necrocytosis (20). To the best of our knowledge, the present study is the first study to show that shikonin promotes autophagy in tumor cells, which may significantly enhance its potential as an antitumor agent. However, the underlying molecular mechanism requires further investigation, as the promotion of autophagy is a complex process.
